# Medical professionalism in ophthalmology: design and testing of a scenario based survey

**DOI:** 10.1186/s12909-020-02071-y

**Published:** 2020-05-19

**Authors:** Eman Alkahtani, Abdullah Assiri, Saba Alrashaed, Mosa Alharbi, Saeed Almotowa, Rajiv Khandekar, Deepak P. Edward

**Affiliations:** 1 The Eye Consultant Clinic, Riyadh, Saudi Arabia; 2 King Khaled Eye Specialist Hospital, P.O. Box 7191, Riyadh, 11462 Saudi Arabia; 3 Magrabi Eye Ear & Dental Hospital, Riyadh, Saudi Arabia; 4 Dr. Sulman Alhabib Hospitals, Riyadh, Saudi Arabia; 5 The Eye Consultant Clinic, Jeddah, Saudi Arabia; 6 University of Illinois Eye and Ear Infirmary, Chicago, IL USA; 7 Wilmer Eye Institute, Johns Hopkins University School of Medicine, Baltimore, MD USA

**Keywords:** Professionalism, Medical profession, Ethics, Ophthalmology

## Abstract

**Background:**

Professionalism is hard to quantify but essential in medical practice. We present a survey tool for ophthalmologists that assessed professionalism using case-based scenarios in central Saudi Arabia.

**Methods:**

Ophthalmologists (resident, fellows and consultants) participated in a web-based survey in 2015. Out of 44 attributes related to professionalism, experts selected 32 attributes with validity indices of ≥0.80. To evaluate these attributes, 51 scenario-based questions were developed and included in the survey. For each attribute, participants were given choices of close ended responses: unacceptable (1), probably unacceptable (2), acceptable (3), probably acceptable (4). The attribute score was compared to the gold standard (responses of an expert group). An attribute score was generated and compared among subgroups.

**Results:**

Of the 155 ophthalmologists, responses of 147 ophthalmologists who completed more than 50% of questions were reviewed. Their mean attribute score was 84.1 ± 10.1 (Median 87.1; 25% quartile 78.1; minimum 50; and maximum 100). The variation in attribute score among consultants, fellows and resident ophthalmologists was significant (*P* = 0.008). The variation of attribute score by groups of attributes was also significant (*P* < 0.05). The score for ‘Personal characteristics’ was on a lower scale compared to that of other attribute groups. The variation in the scores for attribute groups; ‘Personal characteristics attribute’ group (*p* < 0.01) and ‘Workplace practices & relationship’ group (*P* = 0.03) for consultants, fellows and residents were significant.

**Conclusions:**

Professionalism among ophthalmologists and those in training was high and influenced by years of experience. The survey tool appeared to show differences in responses to specific professional attribute groups between trainees and consultants. Additional studies with a larger sample size might be helpful in validating the survey as a tool to be used to assess professionalism in graduate medical education in ophthalmology.

## Background

Professionalism is a competency for physicians that is hard to quantify, yet essential in the practice of medicine. Professionalism has been associated with the practice of medicine for several thousand years and can be traced as far back as the Hippocratic oath [1]. Professionalism also entails the acknowledgement and integration of cultural and religious issues which are important for an effective doctor–patient relationship [2].

Different definitions imply that professionalism encompasses a number of different attributes that are combined to identify and define this medical competency. Chandratilake et al. [3] identified 46 professional attributes that could be useful in assessing physicians but also noted that regional and global consensus on the importance of attributes could vary. Several key similarities and differences in professional attributes were noted among regional groups of 584 medical practitioners from Europe, North America and Asia. In their study, twenty-nine attributes achieved global consensus. Contrary to the evidence in the literature, some of the 46 professional attributes that mainly related to the personal well-being of the physician were considered non-essential by all regional groups. Eleven of the professional attributes differed regionally, which may reflect differences in social, economic and cultural backgrounds. The attributes that were selected by Chandratilake et al. [3] formed the basis of the current study.

Although professionalism is a required competency in undergraduate/ graduate medical education and beyond, it is difficult to define and develop quantifiable measures. Professionalism in all regions including the Arab world was mainly assessed at the medical school level rather than during graduate medical education [4–6].

Although professionalism is considered as one of the competencies required for training during an ophthalmology residency program, to the best of our knowledge, attitudes and knowledge of professionalism among ophthalmologists and ophthalmologists-in-training have not been studied in a quantitative manner [7].

We present the outcomes of a professionalism survey developed by the authors that was administered to ophthalmologists at different levels of experience. The primary purpose of the survey was to test its usefulness as a tool for generating information on professionalism. The secondary objective was to determine the level and variation in professionalism among subgroups of ophthalmologists/ophthalmologists in training.

## Methods

In 2015, ophthalmologists and ophthalmologists-in-training at King Khaled Eye Specialist Hospital, Riyadh, Saudi Arabia were invited to participate in the survey. The internal review board at King Khaled Eye Hospital approved this study.

Ophthalmology consultants who were in active practice for at least five years after fellowship training with considerable experience in teaching and clinical activities constituted an expert group that developed the survey using the attributes described by Chandratilake et al. [3] The correct response to questions in the survey was also determined by this group by consensus as described below. Of the 44 attributes, the expert group identified 32 attributes important to ophthalmology with content validity indices ≥0.80. These attributes were used to develop case- based scenarios using a Delphi based selection consensus process [8]. The attributes were grouped into five broad areas (Table 1).

**Table 1:** Attribute groups used in this study of professionalism among ophthalmologists

Group	Attributes	Attribute question number
1-Personal characteristics	Honesty and integrity; Reliability and dependability; Reflective practice	3,4,6,9,17,22,24,27,33
2-Doctors-patient relationships	Respect for Patient autonomy, confidentiality and privacy; Showing compassion; treating patients fairly without prejudice	1,5,7,15,18,19,31
3-Workplace practices and relationships	Being responsible for commissions and omissions; Being accountable for one’s own actions; Working in teams	2,8,10,11,12,13,14 16,20,23,25,26,29,35
4-Socially responsible behaviors	Law-abiding behaviour; Avoidance of substance and alcohol abuse; Making effective use of the available resources	32
5- personal well-being of doctor	Looking after own health and well-being; Being mindful of personal appearance	30

Missing: 21, 28

Some attributes had more than one case-based scenario accounting for more questions than attributes. In addition, after creation of the survey questions, it was realized the placement of a particular question could be placed in more than one attribute group and the decision for those questions to be placed in a particular group was reach by consensus among the expert group. However, in the final analysis after the survey was administered, questions with the highest response rate for an attribute were chosen. A total of 51 scenario-based questions were created and administered. All questions had 4 response options, unacceptable [1], unacceptable [2], acceptable [3], probably acceptable [4]. The expert group discussed all the questions and consensus was reached for one correct answer for each question. The participant’s response was compared to the expert’s response for determining his/her attribute score described below. The survey used is included as Appendix.

A web-based tool was used to administer the survey anonymously. The survey participants included consultants, fellows and residents. The ophthalmology residents were enrolled in a 4-year training program and fellows in a 2-year post residency-training program. The survey did not distinguish between the resident’s year of training and the fellowship year of training nor did it record the years of experience that a consultant had after training. However, it is to be noted the title of a consultant in the hospital was given to individuals who were at least 3 years post fellowship training.

A response rate of 50% or greater of all attribute related questions was the goal.

The data/ responses was transformed into scores and grouped into two categories in order to simplify analysis [Category 1 = unacceptable + probably unacceptable responses; Category 2 = acceptable + probably acceptable responses]. These responses were then compared with the expert panel consensus for each question and coded as “1” if in agreement with the panel and “0”, if not.

The average score per attribute for each respondent was calculated as:

Mean Attribute Score= $\frac{\mathrm{Total}\ \mathrm{Score}\ \mathrm{Attribute}\ }{\mathrm{Attribute}\ \mathrm{questions}\ \mathrm{answered}}$ X 100.

A higher mean attribute score indicated that the responses of the participant were closer to the consensus of the expert consensus panel.

Responses were analyzed using Statistical Package for Social Sciences (SPSS 23 IBM, NY, USA). Nonparametric tools were used to detect differences in the perception of respondents compared to the expert group for each category and for each attribute group. Results were presented in frequencies and percentage proportions for three groups (consultants, residents, and fellows) and were compared using the Chi-Square test with a 5% level of significance. Descriptive statistics were calculated, and correlation analysis was performed.

The scores among 3 groups (consultants, fellows and residents) were compared with the Freedman test at 5% level of significance. Using nonparametric method with the Mann Whitney U test using a 0.05 level of significance performed additional pairwise comparisons.

Gr 4 has only one attribute (No 32). Number and percentage were calculated for consultant, residents and fellows. Few of them had responded by using nonparametric method chi square value, degree of freedom and two sided *P* value were calculated using SPSS. The variation in these three group was statistically significant.

## Results

The professionalism questionnaire was sent to 155 consultant ophthalmologists/fellows/residents (25 Consultants, 46 fellows and 84 residents).

We analyzed the data from 147 participants (94.8%) who completed more than 50% of the attribute-related survey questions. They included 25 consultants, 42 fellows and 80 residents. The mean attribute score for the expert consensus group which was the standard against which other groups were compared was 100%. The mean attribute score of all respondents was 84.1 ± 10.1 (Median 87.1; 25% quartile 78.1; minimum 50; and maximum 100) (Table 2).

**Table 2:** Response rates by seniority of ophthalmologists

Response rate to survey questions	Consultants (*n* = 25)		Fellows (*n* = 46)		Residents (*n* = 84)		Total
	Number	%	Number	%	Number	%	
> 75%	22	88	33	71.7	55	65.5	110
50 to 74%	2	8	10	21.7	25	29.7	37
< 50%	1	4	3	6.5	4	4.8	8

**Fig. 1:**
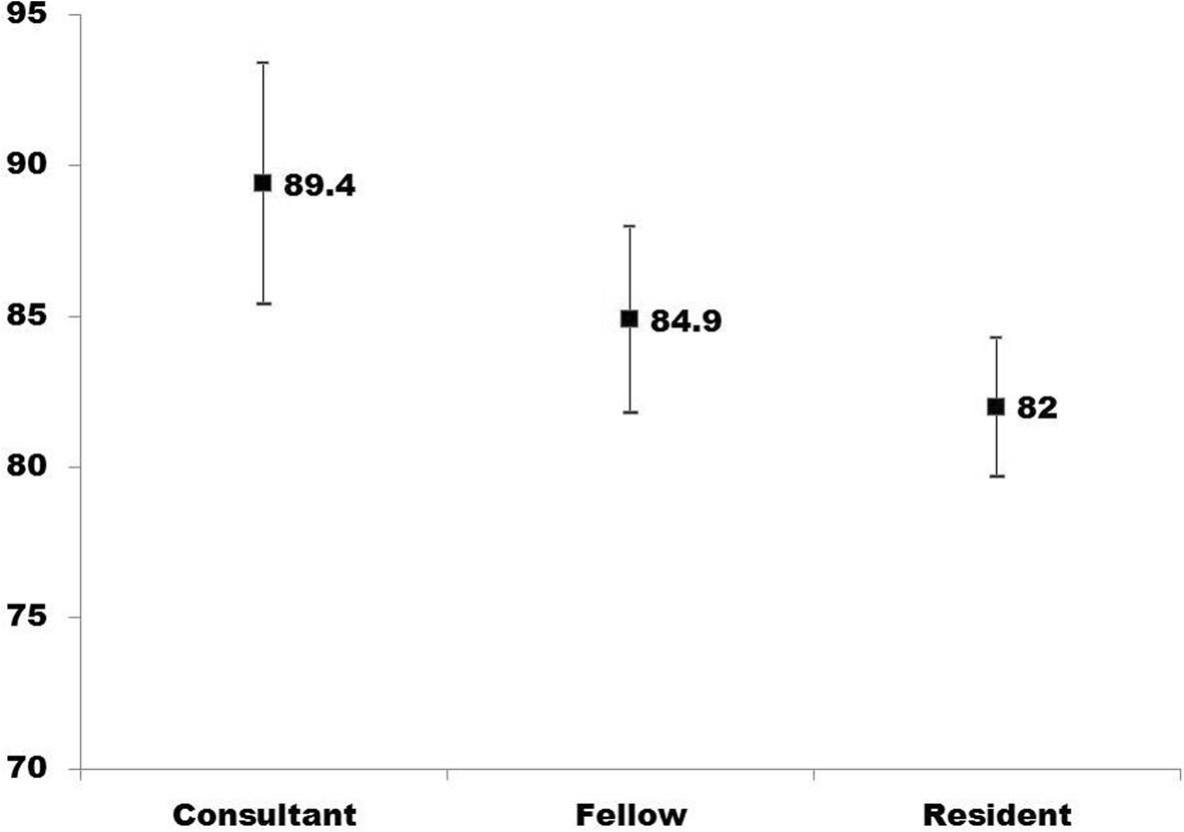
Attribute scores of consultants, fellows and residents based on survey responses. X axis shows experience level of the ophthalmologist. Y axis shows attribute score of the group. The upper and lower end of the line denotes quartiles while central marker shows the median score

We then compared the mean attribute scores of consultants, fellows and ophthalmology residents. There appeared to be a graded difference in the mean attribute scores in the groups studied. The variation in the attribute score among three groups of participants was statistically significant (*P* = 0.008). It should be noted that the mean attribute score of the consultant participants was lower than the expert consensus group (89.1 vs. 100). The mean attribute score of fellows and resident trainee was not significantly different. (*P* = 0.97) (Fig. 1).

**Fig. 2:**
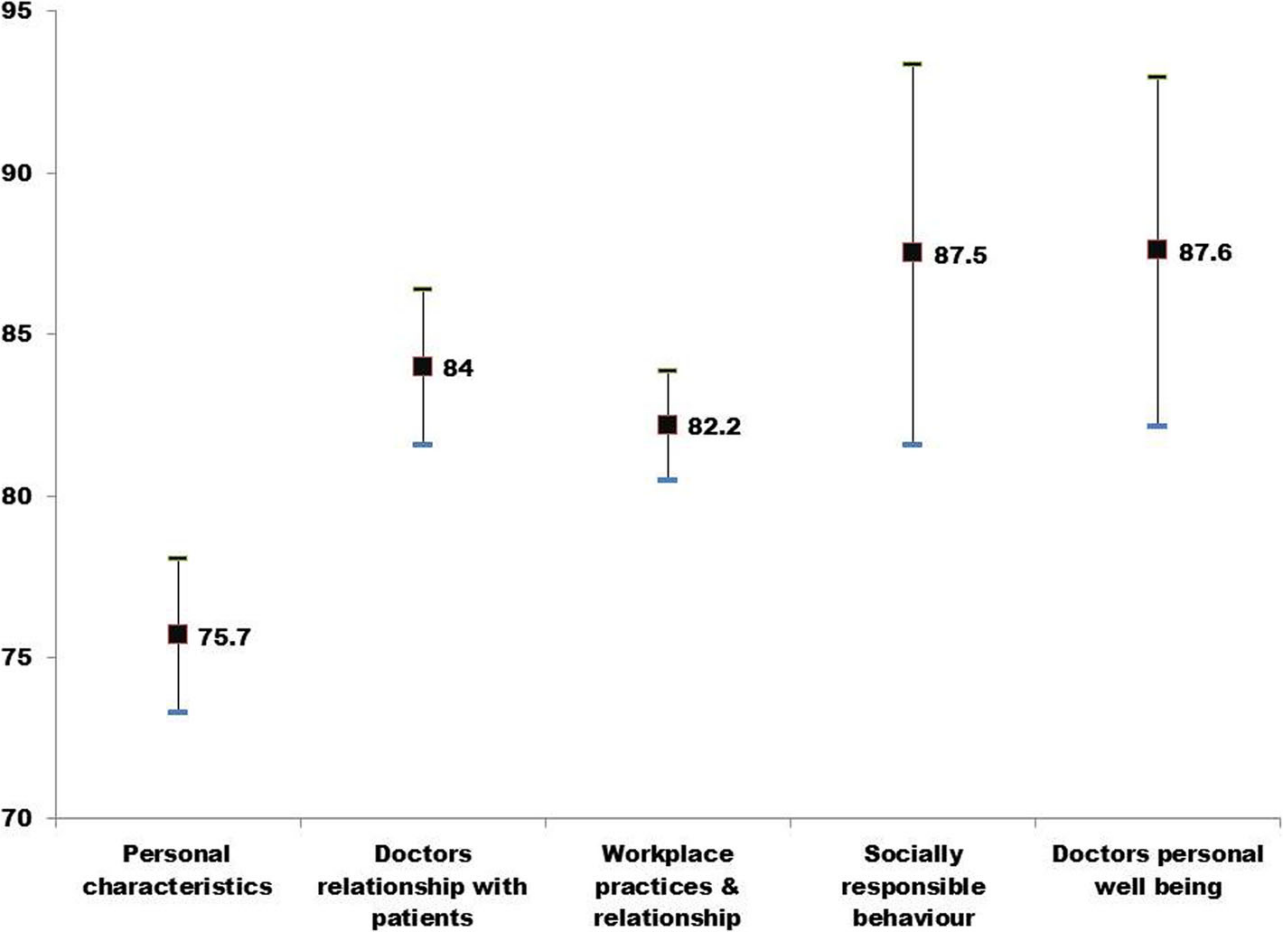
Attribute score of all participants by the category of professionalism attribute. X axis shows category of professionalism attribute. Y axis shows attribute score of the group. The upper and lower end of the line denotes quartiles while central marker shows the median score

As described previously, professionalism related questions fit into five broad categories. The mean attribute score of all ophthalmologists by category of attribute is shown in Fig. 2. Although, all respondents scored high in each of the five attribute categories, scores for the attribute category ‘personal characteristics’ (G1) was lower compared to the scores for other attribute categories. The mean and median attribute scores were lowest in the residents and highest among the consultant ophthalmologists. The mean difference attribute score by category of attribute among the three groups was also significant (*P* < 0.001).

Furthermore, we also compared attribute score by group of attributes among ophthalmologists (Table 3). The variation of attribute score by the attribute group among ophthalmologists (consultants, fellows and residents) was significant (P < 0.001). The differences in the mean scores for categories “Personal characteristics attributes” (G1) (*p* < 0.01) and “Workplace practices & relationship” (G3) (*P* = 0.03) among residents, fellows and consultants were significant. While the difference in the mean score for categories “Doctors’ relationships with patients” (G2) (p 0–099), “Socially Responsible Behaviors” (G4) (p 1.0) and “Doctors’ personal well-being” (G5) (*p* = 0.435) (Table 4).

**Table 3:** Attribute score of 147 ophthalmology resident’s fellows and consultants

All attributes	n	Mean	SDV	Median	25% quartile
Consultant	25	89.4	9.6	90.6	87.5
Fellow	43	84.9	10.3	87.5	80.0
Resident	79	82.0	10.4	84.4	78.1

**Table 4:** Attributes scores among all respondents by category of attribute

Attributes	Mean	SDV	Median	25% quartile	Validation
Group1	75.7	14.2	75.0	62.5	chi square = 176 DF = 4 P < 0.001
Group 2	84.0	13.4	85.7	71.4	
Group 3	82.2	9.4	85.7	78.6	
Group 5	87.6	29.3	100	100	
Group 4	104 (70.7%)				

We also compared the scores within the group of attributes for three levels of seniority (Table 5).

**Table 5:** Attribute score of each group of seniority by category of attributes

Category of attributes			Validation Friedman P
Gr 1	Median IQR	75.0 62.5; 87.0	Friedman P < 0.001
Gr 2	Median IQR	85.7 71.4; 100	
Gr 3	Median IQR	85.7 78.6; 85.7	
Gr 4		–	–
Gr 5	Median IQR	100 100; 100	
Friedman P			

There were similarities and differences between consultants and ophthalmologists in-training in understanding professionalism. The variation in the attribute score among three levels of ophthalmologists was statistically significant (*P* = 0.008).

## Discussion

In the present study, we could quantify and discriminate levels of professionalism between ophthalmologists-in-training, consultants and a group of experts by using a customized scenario-based survey on professionalism. The cutoff of 50% of survey responses found acceptable for analysis in our study was also used by Ho et al. [5] in a study that assessed professionalism.

The United States Accreditation Council for Graduate Medical Education (ACGME) defines competency in professionalism as demonstrating [1] compassion, integrity, and respect for others [2]; responsiveness to patient needs that supersedes self-interest [3]; respect for patient privacy and autonomy [4]; accountability to patients, society, and the profession; and [5] sensitivity and responsiveness to a diverse patient population [9, 10]. Our survey addressed most of the elements that are included in ACGME definition for professionalism.

Chandratilake et al. [3] identified regional similarities and dissimilarities in the understanding of professionalism among medical practitioners that were attributed to cultural differences. Since our study was based on selection of attributes related to professionalism from this study, a comparison of results from the current study to those outside the Arab world should be done with caution.

In this study, a fairly high level of professionalism (based on mean attribute scores) was noted among ophthalmologists and ophthalmologists in training. Chandratilake [ 3], who studied other groups of physicians, also made this observation’. It remains to be determined if this level of professionalism is widespread and whether the results reflect the ability of the survey tool to evaluate professionalism’ in a quantitative manner. These questions could be answered by replicating such a study at other institutions in the region with similar resources and goals.

Different tools have been used to assess and teach professionalism. For example, one method to teach professionalism has been role modeling by mentors/teachers [11]. The high level of professionalism among trainees in our study reflects positively on the professionalism of the consultants that usually are role models for the trainees.

The level of professionalism within a group of physicians can be influenced by many personal and environmental factors [12]. One factor among nurses that correlated with a high degree of professionalism was membership in professional organizations [13]. All consultants and most of the ophthalmologists-in-training that participated in this study were members of local or international professional organizations. It is possible that membership might have influenced the high level of professionalism observed in this study.

In our study, the level of professionalism based on some attributes differed significantly among trainees and consultants. However, professionalism of fellows and resident ophthalmologists was similar. This difference in professionalism could be explained by the difference in the years of professional experience among the study participants which was also noted among pediatricians in the United States [10]. In this study, we did not take into account the postgraduate year of training of the trainees or the number of years that the consultants had been practicing. It remains to be determined if the survey tool could differentiate levels of professionalism based on the number of years of experience during training and beyond. We believe that these attributes are likely to improve with time. A longitudinal prospective study is recommended to study if professionalism attributes improve with time among ophthalmologists-in-training. We believe that large numbers of participants would be needed in a cross-sectional study to answer this question.

A national survey of physicians in United States developed by the American College of physicians to assess attitudes and behavior by using indicators for each domain showed that physicians agreed with standards of professional behavior promulgated by professional societies [14]. In this study physicians reported a high level of conformance with the attribute of honesty with patients, an observation which was similar in the 3 groups. A similar conformance to honesty was noted in our study.

The attributes of professionalism such as doctor-patient relationship, socially responsible behaviors and personal well-being of the doctor did not vary significantly among trainee and consultant ophthalmologists. These core competencies are learned through didactics, and practical experience of observation during medical school. Physician-patient relationships might be influenced by the culture and belief system in which this relationship occurs [11, 12]. This aspect of professionalism, which touches upon physician -patient relationship in any tool that assesses professionalism, must be taken into account. In our study, the patients, residents and fellows were of a Middle Eastern background whereas the cultural background of the consultants participating in the study was variable, many of them being expatriates. It is interesting to know that despite these differences in cultural backgrounds the responses to case-based scenarios that addressed physician-patient relationships were comparable with high mean attribute scores in the three study groups.

Professionalism attributes such as using professional status for personal gain and leadership qualities showed lower scores among trainees than consultants. For example, the question related to using professional status for personal gain was a scenario where a patient working at the airport was contacted to help with a flight reservation. The trainees were more likely to feel that this practice was acceptable and disciplinary action was not necessary under these circumstances. Furthermore, the response to the scenario that represented leadership qualities had to do with disciplinary action for poor communication. It is possible that junior physicians/residents were not aware of such policies and therefore the responses deviated from the expert group. More attention in assisting residents to develop these competencies is recommended.

Even though workplace practices among residents differed from those of consultants there were marginal differences in attributes such as acting in a responsible fashion toward colleagues, following professional rules and regulations, fair treatment of colleagues, and using resources effectively. However, residents disagreed with the experts regarding a positive attitude towards professional development. Perhaps lack of experience or limited understanding of the concept of professional development could be the reason for this observation.

Responses towards conforming to social norms of the participants were statistically significantly different from the experts. The scenarios related to this attribute were related to clothing and a patient requesting to be seen by a physician of the same gender. The reason for this differences in responses between experts and trainees could be cultural and influenced by number of factors. It however highlights the fact that responses and expert opinion with such questions would be dependent on the region where such a question may be used.

The completion of the survey related to the case-based scenarios in the present study was less than desired. The fairly long survey questionnaire could have played a role in this limitation. It is possible that widening the grading response on the Likert scale might have further teased out differences in the groups. However, we believe providing additional choices of response would have resulted in survey fatigue, further reduced the response rate and introduce errors. On solution to this might be to split the survey tool into multiple mini surveys to gauge the level of professionalism. Furthermore, we acknowledge the limitation related source bias and comparison with other published studies. The relationship between participation rate and quality of responses in such a survey is debatable [15]. The ophthalmologists in training were likely to be more technically savvy and possibly more inclined to respond to the web-based surveys compared to consultants who are well versed with conventional surveys. Future surveys could be shorter with an option of being paper based. Furthermore, we did realize that categorization of some of the survey questions to a particular attribute was subjective and based on expert consensus, and in some instances the opinion of the expert group varied on placement of the question under a particular attribute and consensus for these questions was reached based on opinion of the majority. For future surveys that may use these case based scenarios it may be useful to revisit categorization of some of these questions since the subjective assessment of attributes may be influenced by regional opinion.

An information session to explain attributes and importance of a high response rate and completion of survey is recommended prior to administering such a survey to address this weakness noted in our study.

Though not done as a follow-up in this study, we also suggest that following administration of the survey, a debriefing session discussing appropriate responses and the rationale behind them be conducted [16]. The debriefing session might serve as an educational tool for participants, and discussion might lead to further improvement modification in the survey tool.

We believe this paper addresses the development of a professionalism tool which at a later stage can be further validated using 360-degree feedback with the feedback questionnaire having professionalism addressed and correlating the results obtained with the survey tool. In addition, we also noted that the survey has spelling and grammatical errors that should be corrected before it is used in the future.

## Conclusions

A customized survey tool demonstrated a high level of professionalism among ophthalmologists and ophthalmologists in training at a tertiary training institute. The survey tool was able to detect certain differences in professional attributes based on the level of professional experience. Replicating such a survey but with a higher response rate in another training institute will further confirm the usefulness of this tool. If validated, the survey tool can be used as a quantitative assessment tool that will highlight the strengths and weakness in professionalism.

## Data Availability

The data will be submitted with manuscript. We recognize it is not always possible to share research data publicly, for instance when individual privacy could be compromised, and in such instances data availability should still be stated in the manuscript along with any conditions for access.
